# Cartilage‐targeting ultrasmall lipid‐polymer hybrid nanoparticles for the prevention of cartilage degradation

**DOI:** 10.1002/btm2.10187

**Published:** 2020-09-10

**Authors:** Xiangzhao Ai, Yaou Duan, Qiangzhe Zhang, Derrick Sun, Ronnie H. Fang, Ru Liu‐Bryan, Weiwei Gao, Liangfang Zhang

**Affiliations:** ^1^ Department of NanoEngineering Chemical Engineering Program, and Moores Cancer Center, University of California San Diego La Jolla California USA; ^2^ Department of Medicine University of California San Diego La Jolla California USA

**Keywords:** intracartilage delivery, lipid‐polymer hybrid nanoparticle, nanomedicine, osteoarthritis, ultrasmall nanoparticle

## Abstract

Current drug delivery approaches for the treatment of cartilage disorders such as osteoarthritis (OA) remain inadequate to achieve sufficient drug penetration and retention in the dense cartilage matrix. Herein, we synthesize sub‐30 nm lipid‐polymer hybrid nanoparticles functionalized with collagen‐targeting peptides for targeted drug delivery to the cartilage. The nanoparticles consist of a polymeric core for drug encapsulation and a lipid shell modified with a collagen‐binding peptide. By combining these design features, the nanoparticles can penetrate deep and accumulate preferentially in the cartilage. Using MK‐8722, an activator of 5′‐adenosine monophosphate‐activated protein kinase (AMPK), as a model drug, the nanoparticles can encapsulate the drug molecules in high capacity and release them in a sustained and controllable manner. When injected into the knee joints of the mice with collagenase‐induced OA, the drug‐loaded nanoparticles can effectively reduce cartilage damage and alleviate the disease severity. Overall, the ultrasmall targeted nanoparticles represent a promising delivery platform to overcome barriers of dense tissues for the treatment of various indications, including cartilage disorders.

## Graphical abstract

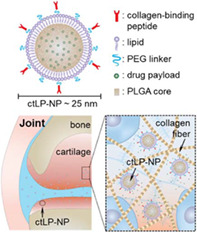

## INTRODUCTION

Osteoarthritis (OA), characterized by progressive degeneration of articular cartilage, chronic pain, and loss of mobility, is the most common joint disorder and the leading cause of disability worldwide.^1^, ^2^ Despite it is an enormous medical need, a disease‐modifying treatment of OA remains unavailable. Managing pain and improving joint function are the current standard of care as the disease progresses.^3^ Although the exact causes of OA remain unknown, a close association of the disease with the aberrant behavior and abnormal phenotype of the resident chondrocytes has been revealed.^4^ As a result, emerging pharmacotherapies are increasingly focused on modulating chondrocyte metabolism, with a hope to halt or reverse OA progression.^5^, ^6^ For this purpose, intra‐articular injection of therapeutic agents is a preferred route of administration.^7^ It offers direct access to the joint space, thereby improving on overall drug bioavailability to the resident chondrocytes while reducing systemic exposure. However, intra‐articular injection of drug molecules is challenged by the fact that subsynovial capillaries and lymphatics can rapidly remove the injected drug from the joint space, and thus the therapeutic benefit is short‐lived.

The pressing needs of improving intra‐articular drug delivery for OA has led to a plethora of nanoparticle designs aimed at enhancing drug retention inside the joint.^8^, ^9^ Compared to small molecules, nanoparticles are more likely to be trapped in the joint space and therefore act as reservoirs for prolonged drug release to the synovial tissue. Despite the advantages, this approach does not assure efficient penetration of drugs into the dense extracellular matrix (ECM) of the cartilage to reach chondrocytes in the deep regions of the cartilage. To address this challenge, adding cartilage‐targeting ability to the nanoparticles has been an attractive strategy to facilitate nanoparticle penetration into the cartilage for high intratissue drug concentration.^8^ In this regard, positively charged nanoparticles have become popular as they spontaneously bind with negatively charged glycosaminoglycans (GAGs) chains of the ECM through electrostatic interactions.^10^, ^11^ Meanwhile, targeting moieties such as anticollagen antibodies and collagen‐binding peptides have been conjugated onto nanoparticles for active targeting to the ECM.^12^, ^13^ Alternatively, nanoparticles have also been made to bind with the resident chondrocytes for targeting. In this regard, synthetic ligands such as chondrocyte‐affinity peptides and natural ligands from the plasma membranes of the immune cells have been leveraged.^14^, ^15^

As researchers continually develop nanoparticles for cartilage targeting, recently, “ultrasmall” lipid‐polymer hybrid nanoparticles (denoted “LP‐NPs”) gained attention for drug delivery.^16^ In this development, charged groups were introduced to the polymer backbones to reduce polymer‐water interfacial tension during nanoprecipitation, resulting in nanoparticles smaller than 30 nm.^17^ Despite their “ultrasmall” sizes, they held a high capacity for encapsulating hydrophobic molecules without burst release. When applied to cancer drug delivery, their ultrasmall size allowed for deep penetration into the dense interstitial space of the tumor microenvironment, and the tumor‐binding ligand conjugated onto the nanoparticle surface reduced their outflux. These distinct features allowed ultrasmall LP‐NPs to deliver a high concentration of anticancer drugs to the tumor, leading to a better antitumor efficacy compared to nontargeted control nanoparticles.

**FIGURE 1:**
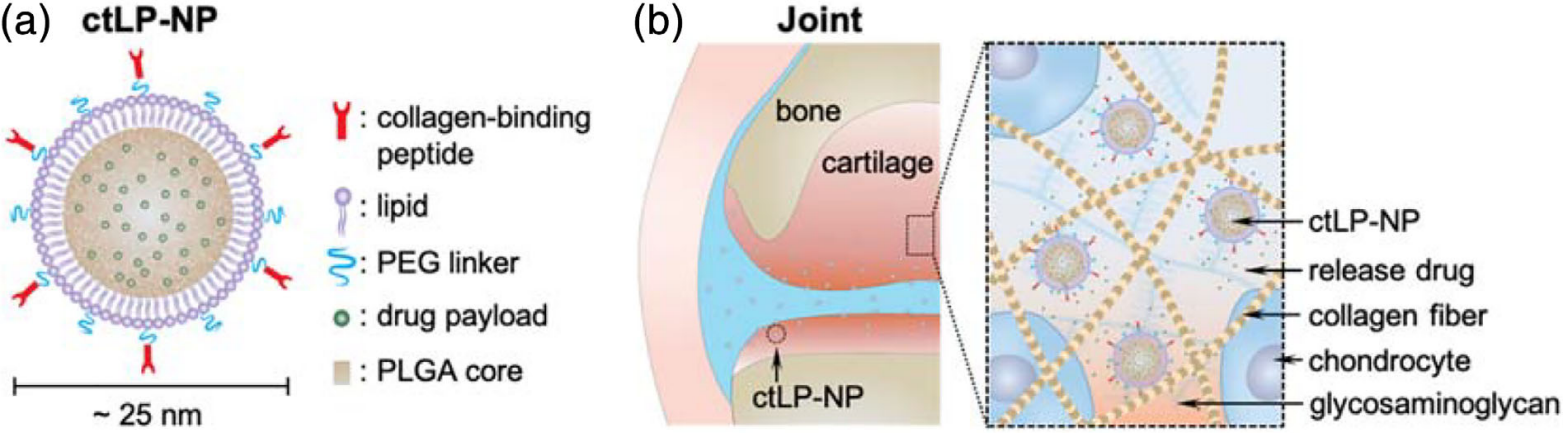
The design of collagen‐targeting ultrasmall lipid‐polymer hybrid nanoparticles (denoted “ctLP‐NPs”) for targeted drug delivery to the joints. (a) Schematic of ctLP‐NPs containing a core made from poly(lactic‐co‐glycolic acid) (PLGA) and a shell made from polyethylene glycol (PEG)‐conjugated lipid. For cartilage targeting, the nanoparticles are modified with collagen‐binding peptides. Hydrophobic drug molecules are encapsulated inside the PLGA cores for delivery. The nanoparticles have a small size around 25 nm. (b) The ctLP‐NPs are able to penetrate deep into the cartilage for effective drug targeting to the chondrocytes

The unique capabilities of ultrasmall LP‐NPs inspire us to apply this nanodelivery platform for drug targeting to the cartilage. Studies have shown that nanoparticles below 40 nm are more likely to penetrate the cartilage than larger nanoparticles.^18^, ^19^ In the current study, we engineered ultrasmall LP‐NPs with a diameter around 25 nm consisting of a core made from poly(lactic‐co‐glycolic acid) (PLGA) and a shell from polyethylene glycol (PEG)‐modified lipid (Figure 1a). With such a small size, the LP‐NPs are expected to penetrate deep into the ECM. We further conjugated the LP‐NPs with a short collagen‐binding peptide (WYRGRLC) that allowed for nanoparticle entrapment in the ECM (Figure 1b).^19^ After confirming their enhanced penetration and retention in the cartilage of mouse femoral head, we loaded them with MK‐8722, a potent activator of 5′‐adenosine monophosphate‐activated protein kinase (AMPK) known to regulate chondrocytes energy metabolism in the cartilage and alleviate OA severity.^20^, ^21^ In the study, the collagen‐targeting LP‐NPs (denoted “ctLP‐NPs”) exhibited sustained drug release over 48 h without drug bursting. When injected into the knee joints of the mice with collagenase‐induced OA, they significantly alleviated the OA severity. Overall, we demonstrated that ultrasmall ctLP‐NPs are a promising nanodelivery platform for cartilage‐targeted drug delivery.

## RESULTS AND DISCUSSION

**FIGURE 2:**
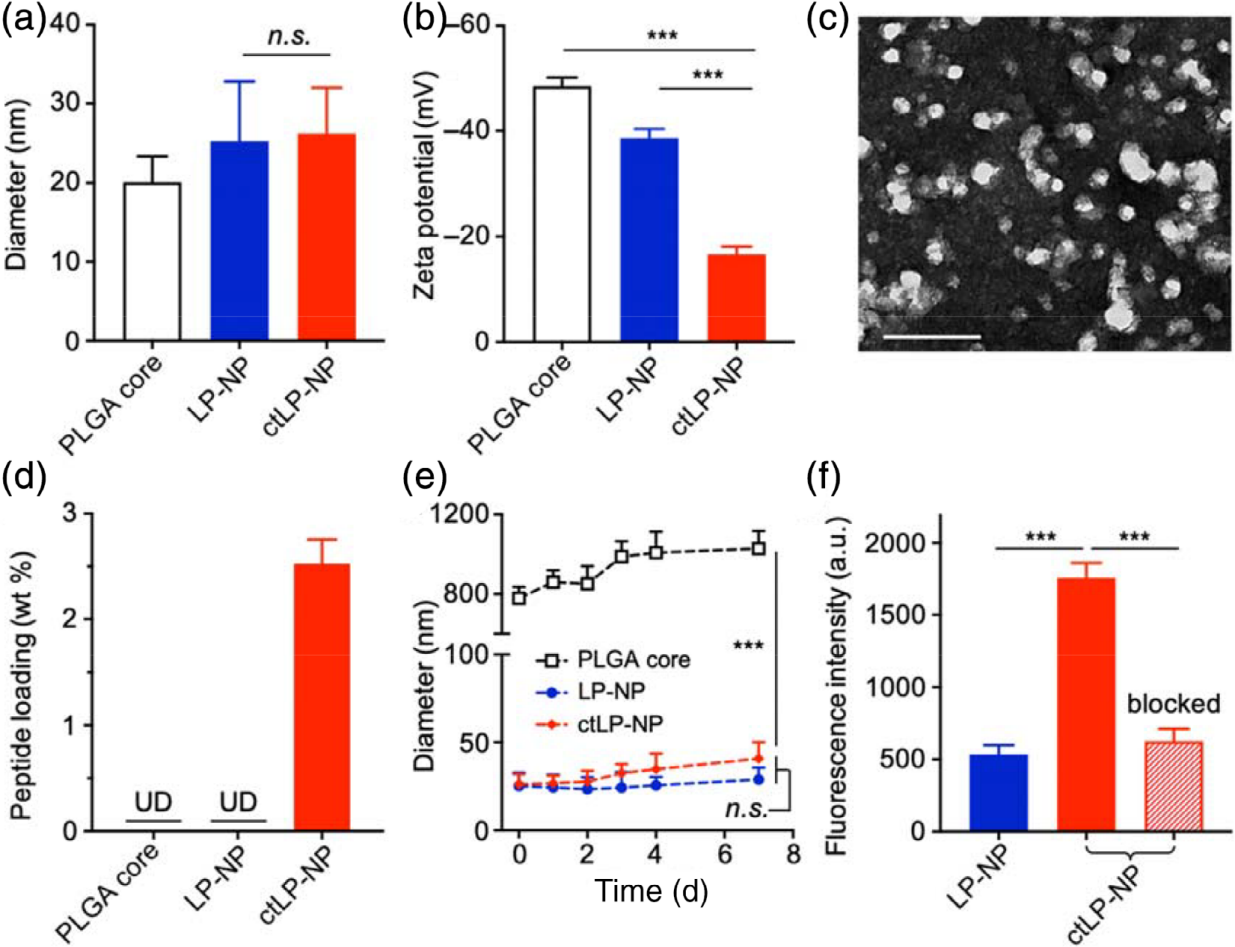
Characterization of ctLP‐NPs. (a) Hydrodynamic size (diameter) of PLGA cores, LP‐NPs, and ctLP‐NPs, respectively. (b) Zeta potential (mV) of different nanoparticle groups. (c) A representative TEM image of ctLP‐NPs with uranyl acetate staining (scale bar, 50 nm). (d) Quantification of protein content on PLGA cores, LP‐NPs, and ctLP‐NPs, respectively, using a BCA assay (UD, undetectable). (e) The hydrodynamic size of PLGA cores, LP‐NPs, and ctLP‐NPs in 1× PBS over a week. (f) Fluorescence intensity of DiD‐labeled LP‐NPs and ctLP‐NPs bound onto a type II collagen‐coated plate. As an additional control (labeled with “blocked”), the plate was pretreated with free peptide to block the collagen before adding ctLP‐NPs. Data presented as mean ± SD (*n* = 3); *n.s*.: not significant; ***p < 0.001; statistical analysis by one‐way ANOVA

In the study, the ctLP‐NPs and nontargeted control nanoparticles (LP‐NPs) were fabricated through a charge‐based nanoprecipitation procedure.^16^ Specifically, carboxylic acid‐terminated PLGA in acetonitrile was added into a Tris–HCl buffer (pH = 8) containing PEG‐lipid molecules, which self‐assembled to form nanoparticles. Collagen‐binding peptides were conjugated to PEG‐lipid to introduce the targeting function. After purification, the dynamic light scattering (DLS) measurements of LP‐NPs and ctLP‐NPs showed a hydrodynamic diameter of 25 nm (Figure 2a). This value was approximately 5 nm larger than that of the bare PLGA cores, an increase consistent with the previous formulation of similar ultrasmall LP‐NPs.^16^ A similar size between the two groups suggests a negligible impact of the conjugated peptide on the nanoparticle size. Meanwhile, the zeta potential value of both nanoparticle groups was less negative than that of the PLGA cores, likely due to the charge shielding by the PEG‐lipid shell (Figure 2b).^22^ The value of ctLP‐NPs was higher, reflecting the presence of the positively charged targeting peptides on the nanoparticle surfaces. When examined with transmission electron microscopy (TEM), ctLP‐NPs appeared as spheres with an average diameter of approximately 25 nm, consistent with the DLS measurements (Figure 2c). ctLP‐NPs also showed a protein content of about 2.5 wt%, whereas no protein was detected in the control nanoparticles, further confirming the successful peptide modification (Figure 2d). In the stability test, the PLGA cores suspended in 1× PBS aggregated rapidly. In contrast, both ctLP‐NPs and LP‐NPs remained stable over a week, demonstrating their high stability in a buffer solution (Figure 2e). To confirm the targeting function, we added the fluorescence‐labeled nanoparticles to the plates coated with type II collagen. After the removal of unbound nanoparticles, a much higher level of ctLP‐NPs was retained when compared with the nontargeted LP‐NPs, indicating the targeting effect from the peptide modification (Figure 2f). When the plates were pretreated with free peptide, the retention of ctLP‐NPs decreased, further confirming the targeting role of the peptide. Overall, these results confirm a successful formulation of ctLP‐NPs.

**FIGURE 3:**
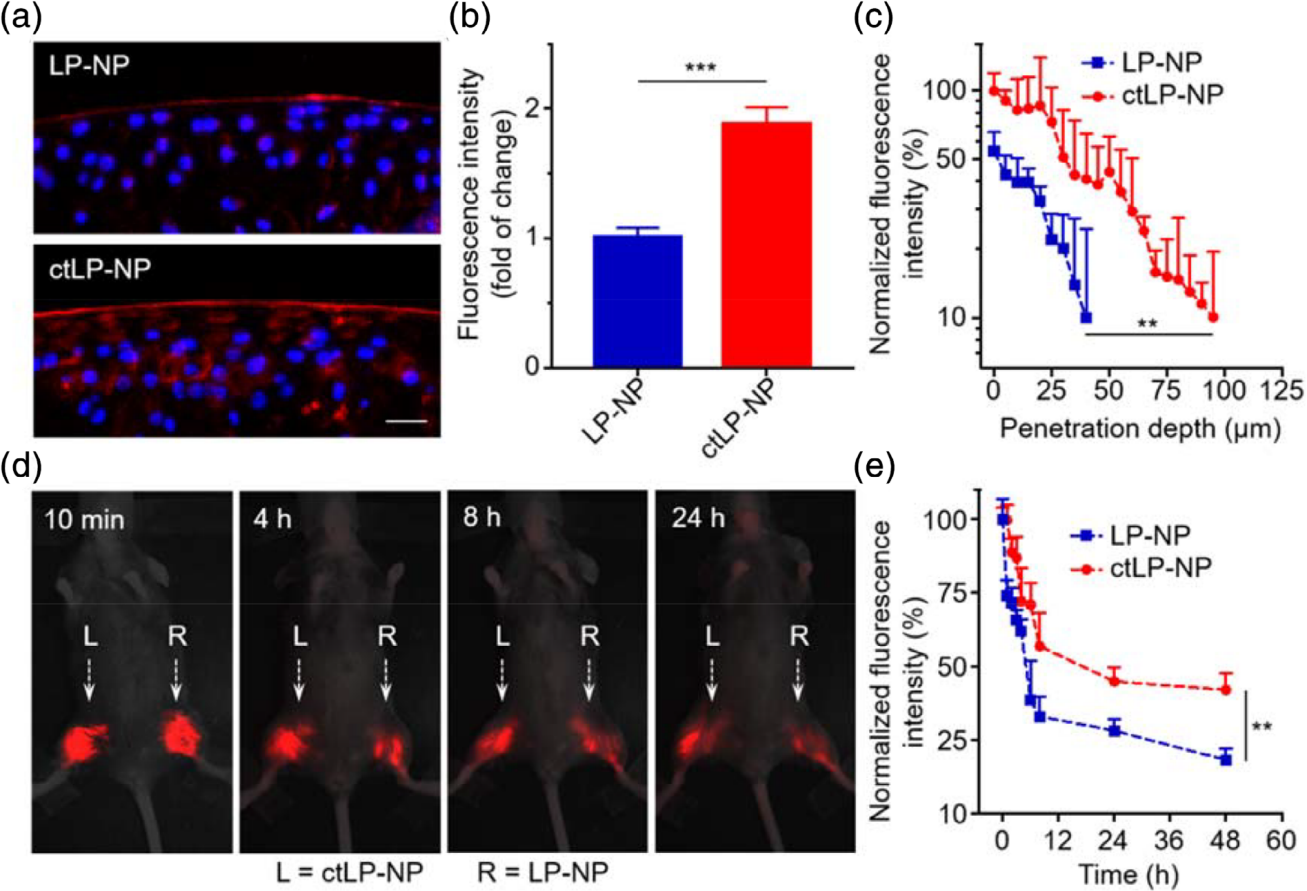
Retention and penetration of ctLP‐NPs in the cartilage. (a) Representative fluorescence images of femoral head sections incubated with DiD‐labeled LP‐NPs (top) or ctLP‐NPs (bottom). Red represents the nanoparticles and blue represents the nuclei (scale bars: 20 μm). (b) Relative fluorescence intensity in the cartilage upon incubation with LP‐NPs or ctLP‐NPs (0.2 mg mL^−1^) for 24 h. (c) Quantitative analysis of nanoparticle penetration depth into femoral heads. (d) Fluorescence images of mouse knee joints after intra‐articular injection of fluorescence‐labeled ctLP‐NPs (left, L) and LP‐NPs (right, R) at different timepoints. In the study, 20 μL of 2 mg mL^−1^ nanoparticle suspension was injected into each joint. (e) Fluorescence intensity of the nanoparticles in the knee joints. Data presented as mean ± SD (*n* = 3). **p < 0.01; ***p < 0.001; statistical analysis by two‐tailed Student's *t*‐test

Next, we examined the penetration and retention of ctLP‐NPs in the articular cartilage of mice. We hypothesize that the ultrasmall size and collagen‐targeting ability together will allow ctLP‐NPs to penetrate deep and stay long in the cartilage. To test this hypothesis, we collected femoral heads from mice and incubated them with DiD‐labeled ctLP‐NPs or nontargeted LP‐NPs in the culture medium. After 24 h, we washed and sectioned the tissue samples for fluorescence microscopic studies. Under the microscope, sections of the articular cartilage incubated with LP‐NPs showed nanoparticle signal at the distal region of the femoral heads (Figure 3a). Sections from the tissue incubated with ctLP‐NPs also showed signals in the same region, but the image appeared brighter, suggesting enhanced retention of ctLP‐NPs in the cartilage. When quantified, the average fluorescence intensity from tissues incubated with ctLP‐NPs showed a nearly twofold increase compared with those incubated with LP‐NPs (Figure 3b). To evaluate cartilage penetration of the nanoparticles, we quantified the fluorescence intensity at different depths by sampling image stacks with a width of 5 μm, and then normalized the value to that of the outermost tissue section. As shown in Figure 3c, the fluorescence signal of LP‐NPs decreased as the depth increased, and the intensity dropped to below 10% when the depth increased to 40 μm. In contrast, the signal from the femoral heads incubated with ctLP‐NPs remained above 10% even when the depth reached 100 μm. We hypothesize that, depending on the nanoparticle size and binding affinity, ctLP‐NP binding with the matrix may lead to a steeper intratissue concentration gradient than that of the nontargeted LP‐NPs. This effect has been shown to boost both penetration and retention.^15^, ^23^

We further examined the nanoparticle retention in vivo by injecting them into the knee joints of mice. As shown in Figure 3d, the fluorescence intensity from both ctLP‐NPs (the left knee, marked with “L”) and LP‐NPs (the right knee, marked with “R”) decreased with time. However, at all timepoints, the joint injected with ctLP‐NPs (“L”) remained brighter than the control joint injected with LP‐NPs (“R”). Further quantification of the fluorescence confirmed the observed differences (Figure 3e). Throughout the study, the signal of ctLP‐NPs remained higher than that of the LP‐NPs. At 48 h, 18% of the LP‐NPs remained inside the knees. In contrast, 42% of the ctLP‐NPs retained. Overall, these results demonstrate significantly enhanced penetration and retention of ctLP‐NPs compared to the nontargeted counterparts in the cartilage of mouse joints.

**FIGURE 4:**
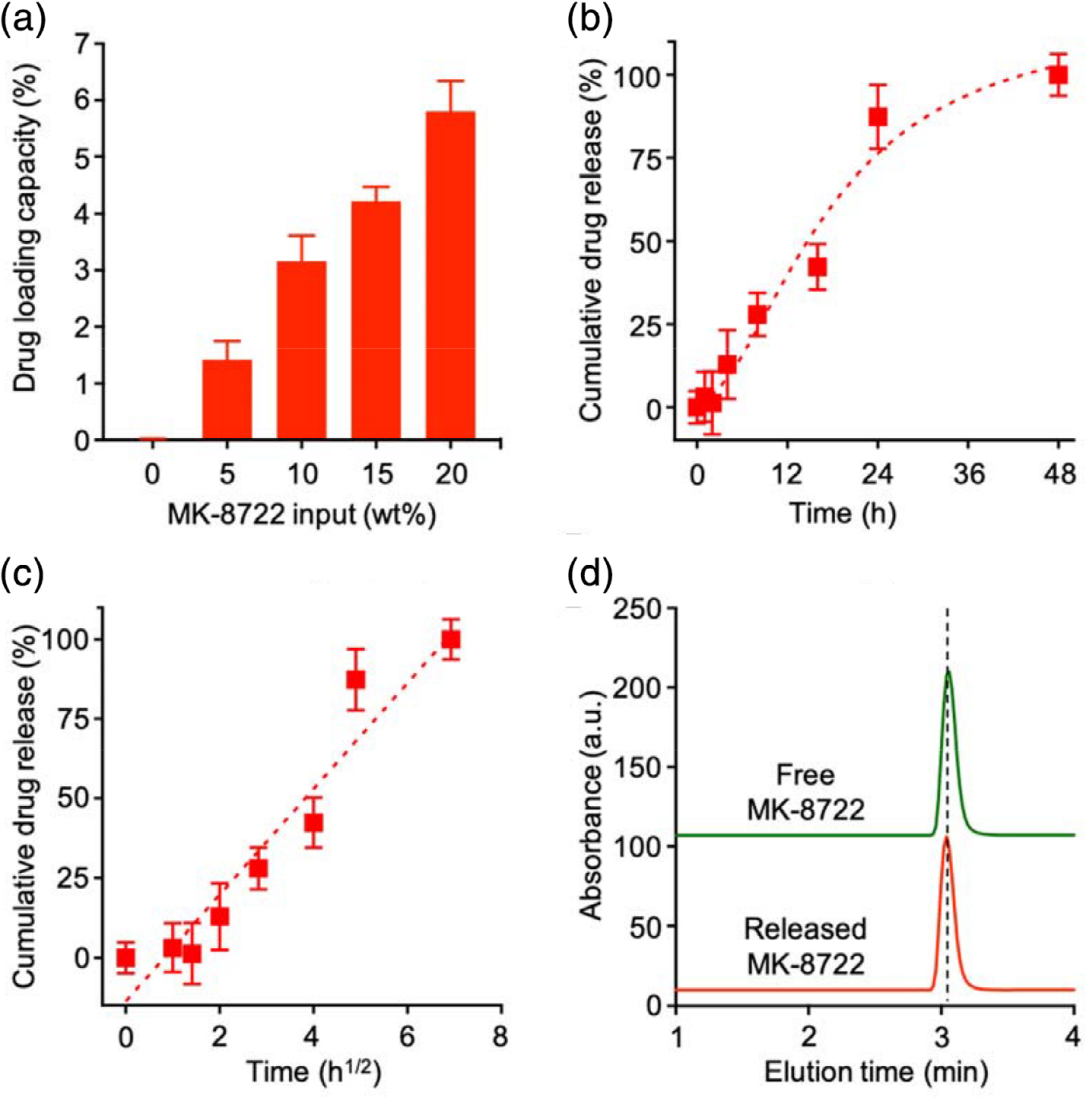
Evaluation of ctLP‐NPs for MK‐8722 encapsulation and release. (a) The relationship between MK‐8722 initial input and its loading capacity. Data presented as mean + SD and *n* = 3. (b) A representative cumulative drug release profile of MK‐8722 from ctLP‐NPs in 1× PBS. (c) A plot of MK‐8722 release percentage from ctLP‐NPs against the square root of the release time. The linear fitting was made by using a diffusion‐dominant Higuchi model. (d) HPLC analysis of free MK‐8722 and released MK‐8722 from ctLP‐NPs, respectively. In (b) and (c), data presented as mean ± SD and *n* = 3

After having characterized the ctLP‐NP formulation, we encapsulated a potent AMPK activator, namely MK‐8722, into the nanoparticles and evaluated the drug loading and release properties.^24^ As a hydrophobic molecule, MK‐8722 can spontaneously incorporate into the PLGA cores during the nanoprecipitation process. To optimize MK‐8722 encapsulation, we tested various drug inputs ranging from 0% to 20% of the total nanoparticle weight. Within this range, drug input had an insignificant impact on the nanoparticle size and their zeta potential (Figure S1). As shown in Figure 4a, the loading capacity of MK‐8722 increased as the initial drug input increased. The initial input of 20% resulted in a drug loading capacity of 5.8 wt%, the highest among all groups. Further increase of the initial input led to observable nanoparticle aggregation and precipitation. Therefore, we chose the drug input of 20% for the following studies. We then investigated the drug release kinetics of the MK‐8722‐loaded ctLP‐NP formulation (denoted “ctLP‐NP(MK)”) in 1× PBS. Overall, we observed a gradual release of MK‐8722: ctLP‐NP(MK) released 74% of MK‐8722 in 24 h and nearly 100% in 48 h (Figure 4b). We further analyzed the release kinetics by using a diffusion‐dominant Higuchi model: *M*_*t*_ = *Kt* ^1/2^, where *M*_*t*_ is drug release at time *t*, and *K* is the Higuchi constant, reflecting the rate at which the drug is released as a function of time.^25^, ^26^ Plotting MK‐8722 release percentage against the square root of time yielded a linear fitting with *R* ^2^ = 0.92 for the ctLP‐NP(MK) (Figure 4c). The goodness of the fit indicates a diffusion‐controlled drug release mechanism. Based on this analysis, the Higuchi constant of the ctLP‐NP(MK) was determined to be 16.7 ± 1.9 h^−1/2^. Moreover, the released drug from ctLP‐NP(MK) showed the same elution time as that of the free MK‐8722 when analyzed with high‐performance liquid chromatography (HPLC), indicating the preservation of drug molecules during the encapsulation and the release processes (Figure 4d). Notably, LP‐NP(MK) showed comparable loading capacity and release kinetics, making it a suitable control group for the following studies (Figure S2).

**FIGURE 5:**
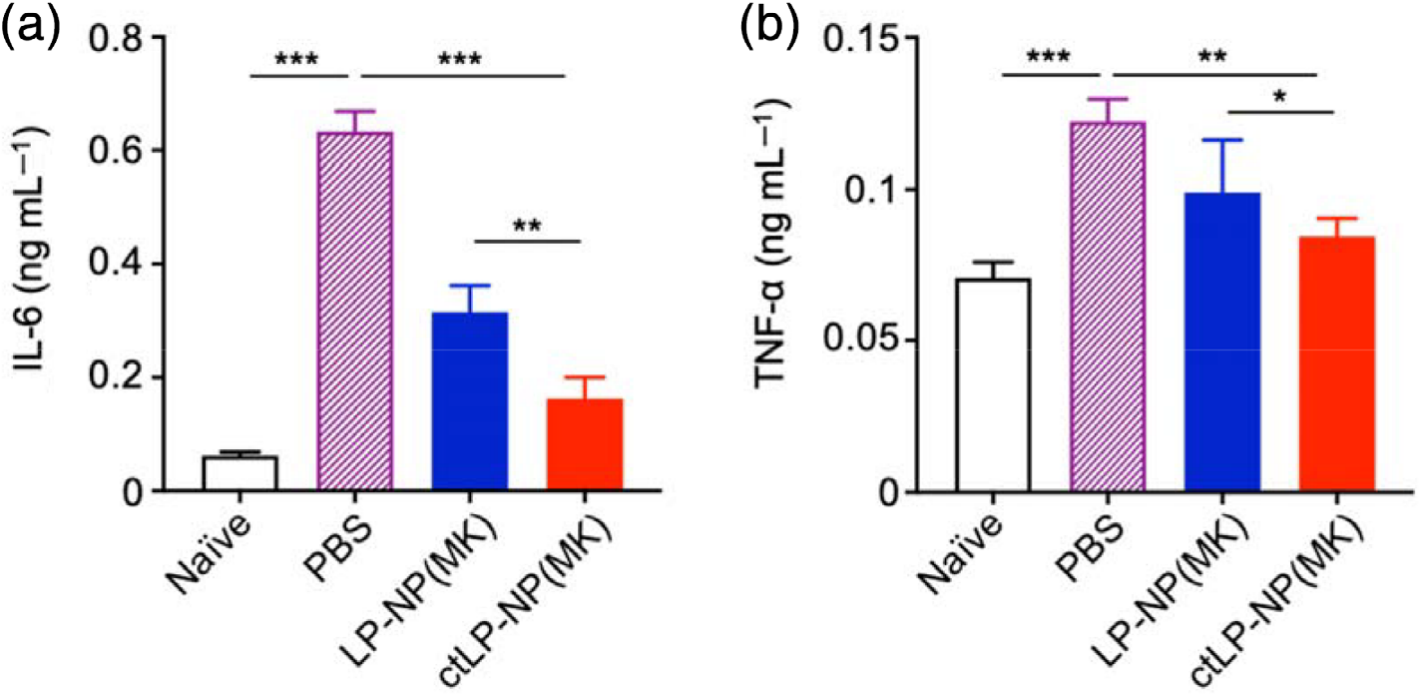
MK‐8722‐loaded ctLP‐NPs [ctLP‐NP(MK)] suppress inflammation in the cartilage. Mouse femoral heads were cultured with IL‐1β (10 ng mL^−1^) to induce inflammation. PBS, LP‐NP(MK), and ctLP‐NP(MK) were added to the tissue samples, respectively. After the incubation, levels of secreted cytokines including (a) IL‐6 and (b) TNF‐α were measured. Data presented as mean ± SD (*n* = 3); *p < 0.05, **p < 0.01, and ***p < 0.001; statistical analysis by one‐way ANOVA

Following the drug encapsulation and release studies, we then examined the efficacy of ctLP‐NP(MK) in alleviating joint inflammation ex vivo. In the study, we collected the femoral heads from mice and stimulated them with IL‐1β (10 ng mL^−1^) mixed with ctLP‐NP(MK). Control groups were stimulated with IL‐1β (10 ng mL ^−1^) mixed with PBS or LP‐NP(MK). At the end of the stimulation, we collected the culture medium and compared levels of IL‐6 and TNF‐α for efficacy. Compared to naïve samples, the femoral heads stimulated with IL‐1β and treated with PBS showed significant production of IL‐6 as the result of the inflammation (Figure 5a). When treated with LP‐NP(MK), the IL‐6 level decreased. The level further reduced to a significantly lower level when treated with ctLP‐NP(MK). Meanwhile, the level of TNF‐α in various groups showed similar changes as that of IL‐6 (Figure 5b). The TNF‐α level in the femoral heads stimulated with IL‐1β and treated with PBS increased significantly compared to that in the naïve sample. However, this level decreased when treated with LP‐NP(MK) or ctLP‐NP(MK). Again, the ctLP‐NP(MK) treated group exhibited the most reduction of TNF‐α among all groups, confirming the benefit of the targeting effect.

**FIGURE 6:**
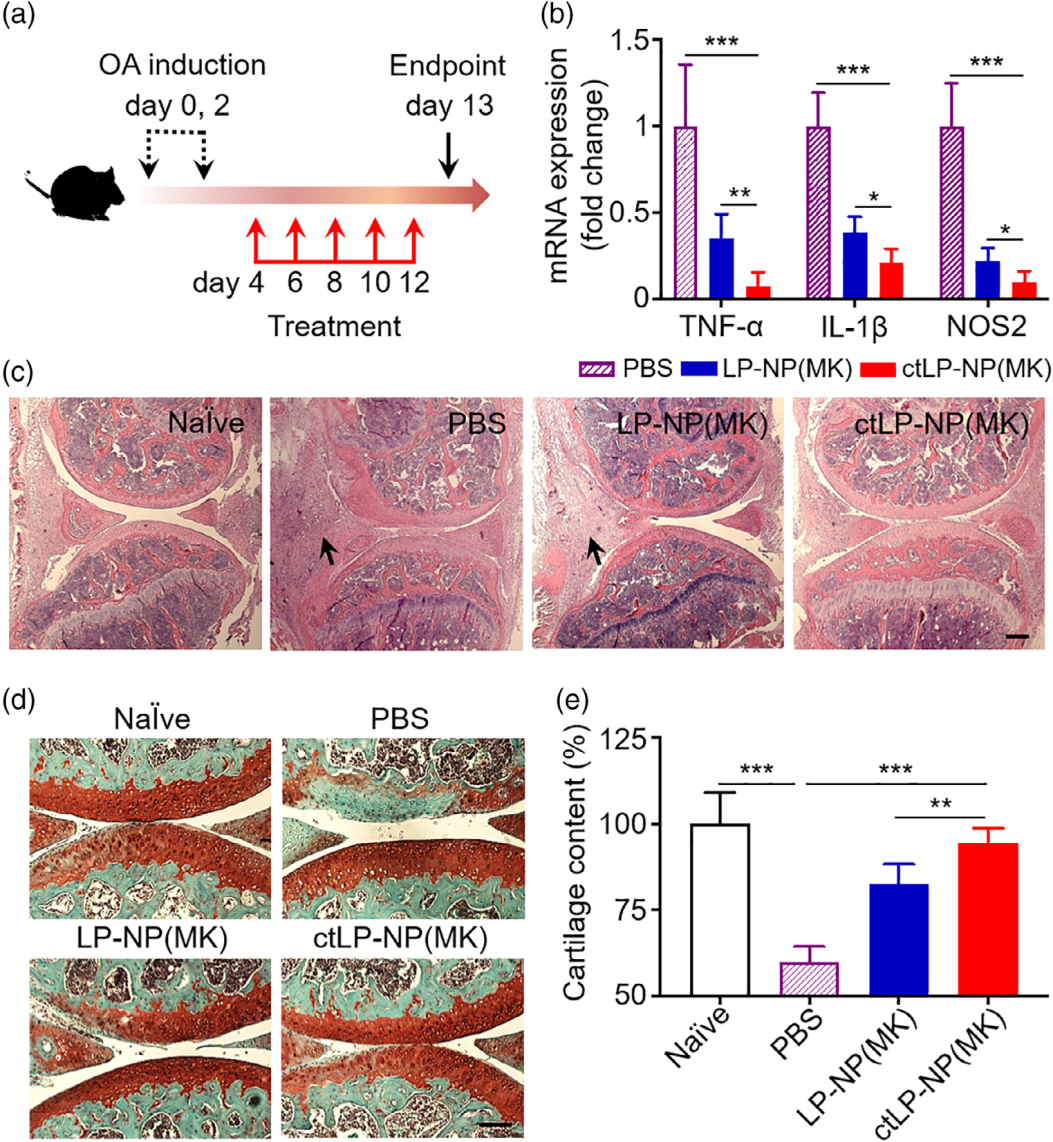
Therapeutic efficacy of MK‐8722‐loaded ctLP‐NPs to repair cartilage damage in the collagenase‐induced OA (CIOA) mice. (a) The study protocol of therapeutic regimen within a CIOA mouse model. (b) Relative mRNA expression of cytokines (TNF‐α, IL‐1β, and NOS2) in the cartilage of mice knee joint upon PBS, LP‐NP(MK), and ctLP‐NP(MK) treatment (intra‐articular injection, 20 μL, 5 mg mL^−1^). (c) Representative images of H&E staining on knee joint sections from healthy mice (Naïve) and CIOA mice treated with PBS, LP‐NP(MK), and ctLP‐NP(MK), respectively. Scale bars, 100 μm. The arrows indicate areas where synovitis is discernible. (d) Representative images of safranin‐O staining on cartilage sections from healthy mice (Naïve) and CIOA mice treated with PBS LP‐NP(MK), and ctLP‐NP(MK), respectively. Scale bars, 100 μm. (e) Quantification of cartilage content from safranin‐O‐stained sections (red) in different groups. Data presented as mean ± SD (*n* = 3); **p < 0.01, ***p < 0.001; statistical analysis by one‐way ANOVA

Lastly, we evaluated the therapeutic efficacy of ctLP‐NP(MK) to reduce inflammation and halt cartilage degeneration in a mouse model of collagenase‐induced OA (CIOA).^27^, ^28^, ^29^ First, we confirmed the OA induction by examining the histopathology of the mouse knees (Figure S3). We then injected PBS, LP‐NP(MK), and ctLP‐NP(MK) (20 μL, 5 mg mL^−1^), respectively, into the knee joints of CIOA mice (every other day, a total of five injections on days 4–12, Figure 6a). During the injection periods, no inflammation and other adverse effects were observed at the sites of injection. Finally, on day 13, we sacrificed the mice and collected their knee joints for analysis. We first assessed the efficacy at a molecular level by evaluating the expression of key pro‐inflammatory cytokines. When tissues from CIOA mice treated with LP‐NP(MK) were analyzed, we found that mRNA levels of TNF‐α, IL‐1β, and nitric oxide synthase 2 (NOS2) were lower when compared to the levels in tissues from mice treated with PBS (Figure 6b). The levels further reduced when mice were treated with ctLP‐NP(MK), revealing an enhanced efficacy via the targeting effect.

We then assessed the efficacy at a tissue level through the histological analysis of the knee sections. When stained with hematoxylin and eosin (H&E), the knee sections from the mice injected with PBS showed signs of synovitis within the synovial tissue (black arrows), including an enlarged synovial lining layer with a higher cellular density and inflammatory infiltrates (Figure 6c). Sections from mice received LP‐NP(MK) showed a moderate enlargement of the synovial lining, suggesting the development of less severe synovitis. In knee sections from mice treated with ctLP‐NP(MK), all indications of the synovitis were absent. The histological features appeared similar to those of the naïve mice, implying a further alleviation of the disease. We further stained the joint sections with Safranin‐O to analyze cartilage histopathology.^30^ The sections from the naïve mice showed a homogeneous cartilage matrix with an intact surface and perichondrium (Figure 6d). In contrast, the sections from PBS‐treated mice showed a partial loss of Safranin‐O staining without cartilage structural damage, indicating cartilage ECM degradation. However, the cartilage content increased in mice treated with LP‐NP(MK). The effects became more apparent in sections from mice treated with ctLP‐NP(MK), where the cartilage and matrix structures were mostly intact compared to those of the naïve mice. Quantification of the cartilage content further confirmed the observation (Figure 6e). The cartilage content in CIOA mice treated with PBS was only 60% of that of the naïve mice. The content recovered to 83% in mice treated with LP‐NP(MK) and reached 95% in those treated with ctLP‐NP(MK).

## CONCLUSION

We developed an ultrasmall lipid‐polymer hybrid nanoparticle system capable of cartilage targeting, efficient drug encapsulation, and controlled release. Because of these unique features, the nanoparticles showed deep penetration and enhanced retention in the dense collagen matrix of the cartilages. Using an AMPK activator as a model drug, we demonstrated their therapeutic efficacy in alleviating cartilage degeneration in a mouse model of collagenase‐induced OA. The design of ctLP‐NP offers attractive features for downstream translation. For example, incorporating the charge‐controlled size reduction into the nanoprecipitation maintains a modular and highly scalable process of fabricating these nanoparticles. Using PLGA cores, the nanoparticles can encapsulate a diverse range of drug compounds targeting existing and emerging pathways in OA. The nanoparticle surface can be readily functionalized with appropriate ligands for targeted delivery. With a lipid‐polymer hybrid design, ctLP‐NP combines the merits of both liposomes and polymeric nanoparticles while overcoming their limitations such as structural instability, content leakage. From a perspective of translational medicine, large‐scale production of lipid‐polymer hybrid nanoparticles has already been developed, paving the way toward future clinical translation.^31^ Despite the promise, cartilage degradation is a complex and multifactorial process. In our model, partial loss of Safranin‐O staining was observed, indicating cartilage ECM degradation. To further assess the beneficial effect of ctLP‐NP on preventing cartilage degradation, the efficacy need to be tested in additional mouse models of OA that emphasize different features of OA pathogenesis.^32^, ^33^ Mechanistic studies to differentiate roles played by chondrocytes, synoviocytes, and immune cells during the treatment will help to further optimize nanoparticle size, ligand density, and drug release profile.^34^ Overall, the targeted ultrasmall nanoparticles hold great promise for effective drug delivery to dense tissues for preventing cartilage degradation. The continuous development of ctLP‐NP may lead to a new treatment modality for OA and other cartilage disorders.

## EXPERIMENTAL SECTION

### Preparation of ultrasmall lipid‐polymer hybrid nanoparticles

The LP‐NPs were prepared by following a previously published procedure.^16^ Briefly, 0.4 mg of 1,2‐distearoyl‐*sn*‐glycero‐3‐phosphoethanolamine‐*N*‐[methoxy(polyethylene glycol)‐2000] (DSPE‐PEG_2000_, Laysan Bio) was dissolved in 10 μL of dimethyl sulfoxide (DMSO). The solution was hydrated by adding 4 mL of Tris–HCl buffer (pH 8, 10 mM) and then sonicated with a bath sonicator (Fisher Scientific FS30D) for 2 min. Then 1 mL of carboxylic acid‐terminated poly(lactic‐co‐glycolic acid) (PLGA‐COOH, 0.67 dL g^−1^, 50:50 ratio, Lactel Absorbable Polymers) in acetonitrile (1 mg mL^−1^) was added rapidly to the DSPE‐PEG_2000_ solution. The mixture was stirred at room temperature for 30 min, leading to the formation of the ultrasmall LP‐NPs. The sample was washed with centrifugal filters (Amicon, Sigma‐Aldrich, with a molecular weight cutoff [MWCO] of 100 kDa). For specific binding to collagen, WYRGRLC peptide (Genscript, 11.2 mg, 12 μmol) was dissolved in DMSO and mixed with 1,2‐distearoyl‐*sn*‐glycero‐3‐phosphoethanolamine‐*N*‐[maleimide (polyethylene glycol)‐2000] (DSPE‐PEG_2000_‐Maleimide, Laysan Bio, 41.2 mg, 14 μmol in DMSO). The final volume of the mixture was adjusted to 1 mL. The conjugation was allowed to proceed for 24 h. Following the reaction, the solution was used to synthesize peptide‐modified ultrasmall LP‐NPs that can target collagen (ctLP‐NPs) by following the same procedure as described above. Bare PLGA core was prepared similarly but without adding lipids to the Tris–HCl buffer. For fluorescence labeling, 1,1′‐dioctadecyl‐3,3,3′,3′‐tetramethylindodicarbocyanine perchlorate (DiD, excitation/emission = 644/665 nm, Thermo Fisher Scientific) was mixed with PLGA in acetonitrile (0.1 wt%) followed by nanoparticle formulation.

### Nanoparticle characterization

The size and surface zeta potential of the nanoparticles were determined by dynamic light scattering (DLS, Malvern ZEN 3600 Zetasizer). For examining the morphology with transmission electron microscopy (TEM), the nanoparticles solution (10 μg mL^−1^) was dropped on a carbon‐coated copper grid (400‐mesh, Electron Microscopy Sciences), washed with DI water, and stained with uranyl acetate (1 wt%, Sigma‐Aldrich). The grid was imaged with an FEI 200KV Sphera microscope. The peptide content on the ctLP‐NPs was quantified using a bicinchoninic acid (BCA) kit (Thermo Fisher Scientific) in reference to a bovine serum albumin (BSA) standard. The stability of bare PLGA cores, LP‐NPs, and ctLP‐NPs were measured by monitoring their sizes in 1× PBS over a period of 1 week.

### Collagen binding study

Collagen (type II, from chicken sternal cartilage, Sigma‐Aldrich) was dissolved in 0.25% acetic acid at a concentration of 0.5 mg mL^−1^. Then 100 μL of the solution was added into each well of a 96‐well assay plate. The plate was incubated at 4°C overnight. Prior to the binding study, the plate was first blocked with 2% BSA for 1 h at room temperature and washed three times with 1× PBS. Then 100 μL of fluorescence‐labeled LP‐NPs or ctLP‐NPs (1 mg mL^−1^ in water) was added to each well and incubated for 1 h with gentle shaking at room temperature. After incubation, the plates were washed with 1× PBS containing 0.05% Tween 20 (National Diagnostics) for three times. Retained nanoparticles were then dissolved with 100 μL of DMSO, and the fluorescence intensity was measured with a Tecan Infinite M200 plate reader.

### Animal use and care

All animal studies were approved under the guidelines of the University of California San Diego (UCSD) Institutional Animal Care and Use Committee. Mice were housed in an animal facility at UCSD under federal, state, local, and National Institutes of Health (NIH) guidelines for animal care.

### Cartilage penetration and retention study

Mouse femoral heads were collected from 10‐week‐old C57BL/6 mice and cultured for 48 h in serum‐free DMEM medium (4.5 g L^−1^ glucose, Hyclone). Then 0.2 mg mL^−1^ of DiD‐labeled LP‐NPs or ctLP‐NPs were incubated with the femoral heads at 37°C for 24 h. After the incubation, femoral heads were washed with 1× PBS three times and embedded in Tissue‐Tek OCT compound (Sakura Finetek) for frozen sectioning. The frozen slices were stained with 4,6‐diamidino‐2‐phenylindole (DAPI, 1 μg mL^−1^, Sigma‐Aldrich) for 30 min, washed with water twice and imaged with an EVOS inverted fluorescence microscope (Thermo Fisher Scientific). Images were analyzed by ImageJ to quantify nanoparticles penetration depth into the cartilage tissues. Briefly, images were extracted from the 5‐μm‐thick sections starting from the distal femoral head surface. Fluorescence of each image section was measured with ImageJ and normalized to the fluorescence signal of outermost section in the femoral heads with different nanoparticles treatment. To study the retention of different nanoparticle formulations in vivo, DiD‐labeled LP‐NPs or ctLP‐NPs (20 μL, 2 mg mL^−1^) was injected intra‐articularly into the bilateral knee joints of C57BL/6 mice. At designated timepoints, mice were imaged with an IVIS system (Fluobeam, Fluoptics). Fluorescence intensities were also analyzed with ImageJ and normalized to the initial fluorescence signal after nanoparticle injection.

### Drug loading and release study

To load the drug, MK‐8722 (AOBIOUS Inc.), a 5′‐adenosine monophosphate‐activated protein kinase (AMPK) activator, was dissolved in DMSO (100 mM) and mixed with PLGA solution, followed by the nanoparticle formulation. Nanoparticles were washed to remove unencapsulated drug molecules with Amicon ultra centrifugal filters (100 kDa MWCO). To quantify the drug loading capacity (calculated by dividing the encapsulated drug by the total nanoparticle weight), washed nanoparticles were dissolved in acetonitrile to release the drug. Samples were analyzed for MK‐8722 with UV absorbance at the wavelength of 315 nm using a Tecan Infinite M200 plate reader. Known concentrations of MK‐8722 were used to generate a standard curve. Different weight ratios of MK‐8722 to PLGA polymer, ranging from 0% to 20%, were tested to maximize the drug loading capacity. Drug release was measured by dialyzing drug‐loaded nanoparticles against 1× PBS in 10 kDa MWCO Slide‐A‐Lyzer MINI dialysis cups (Thermo Fisher Scientific). To measure the amount of drug retained, at predetermined timepoints, samples were collected, lyophilized, and then analyzed with UV‐absorbance as described above. The integrity of released drug was analyzed on a HPLC system (Agilent 1220 Infinity II LC) using a C18 analytical column (Brownlee) with a mobile phase of 50:50 water to acetonitrile containing 0.1% trifluoroacetic acid (TFA). The absorbance wavelength for the detection was 315 nm.

### Mouse femoral head degradation assay

Mouse femoral heads were collected from 10‐week‐old C57BL/6 mice. To study the degradation, 10 ng mL^−1^ of IL‐1β was mixed with 2 mg mL^−1^ drug‐loaded ctLP‐NPs or LP‐NPs in 100 μL DMEM medium and incubated with femoral heads in 96‐well plate to stimulate cartilage degradation for 24 h. The medium was then changed, and the same amount of cytokine–nanoparticle mixture was added to the femoral heads. A total of three treatments, 24 h each, were conducted. Meanwhile, the culture media after the final treatment were collected for measuring the concentrations of IL‐6 and TNF‐α using an enzyme‐linked immunosorbent assay (ELISA, BioLegend).

### Mouse models of osteoarthritis

The CIOA mouse model was established by following a published method.^35^ Briefly, the male C57BL/6 mice (10–12 weeks old) were randomly housed in filtertop cages with 12 h light–dark cycles and fed with a standard diet. CIOA mice were achieved by two intra‐articular injections of 1 U collagenase type VII (Worthington Biochemical) in 10 μL sterile PBS per dose on day 0 and day 2. All the injections were performed in the right knee of the hind legs on the medial side of the knee joint by using a BD ultrafine insulin syringe 29G 1/2′ (BD) under general anesthesia with ketamine (100 mg kg^−1^) and xylazine (10 mg kg^−1^).

### In vivo efficacy study protocol and histological analysis of the knee joints

To study therapeutic efficacy with CIOA mice, 20 μL of drug‐loaded ctLP‐NPs or LP‐NPs (5 mg mL^−1^) in PBS was injected every other day into the knee joint of mice on days 4–12 (after OA induction). As a negative control, PBS was injected. Following the treatment, mice were sacrificed on day 13 to collect hind legs. The knee joint sections were isolated and fixed in 10% formalin for 24 h. Fixed tissues were then dehydrated in 20% sucrose‐PBS for 24 h, and decalcified in 10% EDTA solution for 72 h (solution renewed every day). Processed tissues were sectioned for hematoxylin and eosin (H&E) staining or safranin‐O staining for the cartilage. Sections were counter‐stained with hematoxylin to visualize cell nuclei. Images were taken with a Micromaster II Microscope (Fisher Scientific). The safranin‐O‐positive area was quantified by using ImageJ.

### Quantification of cytokines mRNA expression

The quantitative reverse transcription‐polymerase chain reaction (qRT‐PCR) technique was utilized to determine cytokines gene expression. The knee joints were collected from CIOA mice and dissected to remove extra‐articular tissue. The articular tissues were then homogenized three times using a Biospec Mini Beadbeater in 0.5 mL of RNA lysis buffer for 1 min. The total RNA was extracted from the chondrocytes using Direct‐zol RNA miniprep kit (Zymo Research), and complementary DNA (cDNA) was synthesized using ProtoScript ® First Strand cDNA Synthesis Kit (New England Biolabs) according to the manufacturer's protocol. The qRT‐PCR tests were performed in a QuantStudio 3 Real‐Time PCR System (ThermoFisher Scientific) using the Maxima SYBR Green/Fluorescein qPCR Master Mix kit (ThermoFisher Scientific). The primer sequences are shown as follows (F: forward and R: reverse): IL‐1β (F: 5′‐TGCTGTCGGACCCATATGAG‐3′, R: 5′‐ATCCACACTCTCCAGCTGCA‐3′), TNF‐α (F: 5′‐GGTGCCTATGTCTCAGCCTCTT‐3′, R: 5′‐GCCATAGAACTGATGAGAGGG

AG‐3′), NOS2 (F: 5′‐GGAGTGACGGCAAACATGACT‐3′, R: 5′‐TCGATGCACAACT

GGGTGAAC‐3′), and GAPDH (F: 5′‐GGAGAAACCTGCCAAGTATG‐3′, R: 5′‐GTCA

TTGAGAGCAATGCCAG‐3′). Each sample was measured in triplicate and the fold changes in relative gene expression were calculated using the 2^−ΔΔCt^ method, with GAPDH serving as the housekeeping gene.

## CONFLICT OF INTERESTS

The authors declare no conflict of interest.

## Supporting information


**Data S1**: Supporting Information
