# Supplementary material for: Cartilage‐targeting ultrasmall lipid‐polymer hybrid nanoparticles for the prevention of cartilage degradation
Source: Bioeng Transl Med. 2020 Sep 10;6(1):e10187. doi: 10.1002/btm2.10187 (PMC7823131; doi:10.1002/btm2.10187)
Supplement: Supplementary file 1 — Data S1: Supporting Information [file BTM2-6-e10187-s001.docx]

Supporting Information:

**Cartilage-Targeting Ultra-Small Lipid-Polymer Hybrid Nanoparticles for the Prevention of Cartilage Degradation**

Xiangzhao Ai, Yaou Duan, Qiangzhe Zhang, Derrick Sun, Ronnie H. Fang, Ru Liu-Bryan, Weiwei Gao,* and Liangfang Zhang*

Dr. X. Ai, Y. Duan, Q. Zhang, D. Sun, Dr. R. H. Fang, Dr. W. Gao, and Prof. L. Zhang

Departments of NanoEngineering, Chemical Engineering Program, and Moores Cancer Center, University of California San Diego, La Jolla, California 92093, USA

* Correspondence: [w5gao@ucsd.edu](mailto:w5gao@ucsd.edu) and [zhang@ucsd.edu](mailto:zhang@ucsd.edu)

Prof. R. Liu-Bryan

Departments of Medicine, University of California San Diego, La Jolla, California 92093, USA

**Supplementary Figure 1. Effects of drug input on LP-NP size and surface zeta potential**

To characterize the effect of MK-8722 encapsulation on the physicochemical properties of the resulting nanoparticles, dynamic light scattering (DLS) measurements were conducted on nanoparticles with various drug inputs. Briefly, MK-8722 with different drug-to-PLGA weight ratios, ranging from 0% to 25%, were used to prepare the drug-loaded LP-NPs and ctLP-NPs following the same procedure as described in the experimental section. Size distribution and surface zeta potential were determined by DLS analysis.


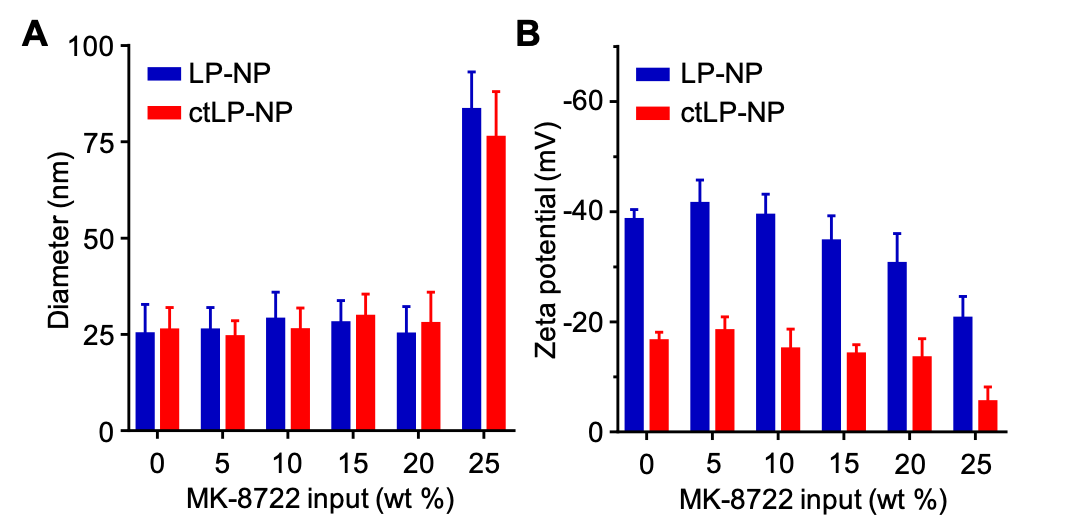


**Figure S1.** DLS measurements of number-based size (diameter) (**A**) and surface zeta potential (**B**) of LP-NPs and ctLP-NPs with increasing MK-8722 input (drug-to-PLGA weight ratios 0 ~ 25 wt%). Data presented as mean + s.d. (*n* = 3).

**Supplementary Figure 2. MK-8722 loading and release properties of LP-NPs**

To optimize the MK-8722 loading capacity, different nanoparticles with various drug inputs were prepared by following the same procedure as described in the experimental section. After drug encapsulation, the nanoparticles were washed with Amicon ultracentrifugal filters (100 kDa MWCO) to remove unencapsulated drug molecules. The drug loading capacity was further quantified by dissolving the nanoparticles in acetonitrile and analyzing the UV absorbance (315 nm) using a plate reader. In the drug release study, samples were collected at pre-determined time points to measure the amount of drug retained in LP-NPs by UV-absorbance. The drug release kinetics was analyzed based on a diffusion-dominant Higuchi model: *M_t_ = Kt^1/2^*, where *M_t_* is drug release percentage at time *t* in hours and *K* is the Higuchi constant.


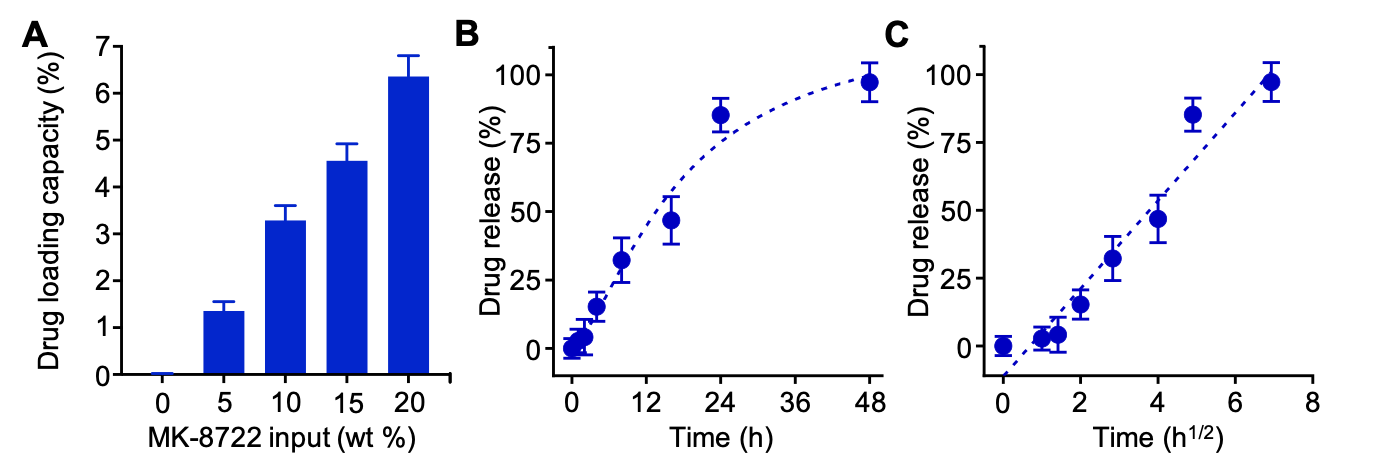


**Figure S2.** Drug loading and release kinetics of LP-NPs *in vitro*. (**A**) Optimization of MK-8722 loading amount in LP-NPs with increasing drug input to PLGA weight ratios. Data presented as mean + s.d. (*n* = 3). (**B**) Representative drug release profile of LP-NPs with MK-8722 input of 20 wt% in PBS for 48 h. (**C**) Plotting of MK-8722 release percentage from LP-NPs against the square root of release time with a linear fitting using a diffusion-dominant Higuchi model. In (B) and (C), data presented as mean ± s.d. (*n* = 3).

**Supplementary Figure 3. Confirmation of OA induction by collagenase**

To establish the collagenase-induced OA (CIOA) mouse model, two intra-articular injections of 1 U collagenase type VII in 10 µL sterile PBS were performed on day 0 and day 2 in the right hind knee joint of healthy C57BL/6 mice (10-12 weeks old). As a negative control, PBS was injected at the same conditions. The mice were sacrificed on day 13 to collect hind legs. The knee joint sections were isolated and processed for safranin-O staining. Images were taken with a Micromaster II Microscope, and the safranin-O-positive area (red) in the cartilage was quantified with ImageJ. The cartilage damage was further scored using the typical osteoarthritis research society international (OARSI) scoring method.


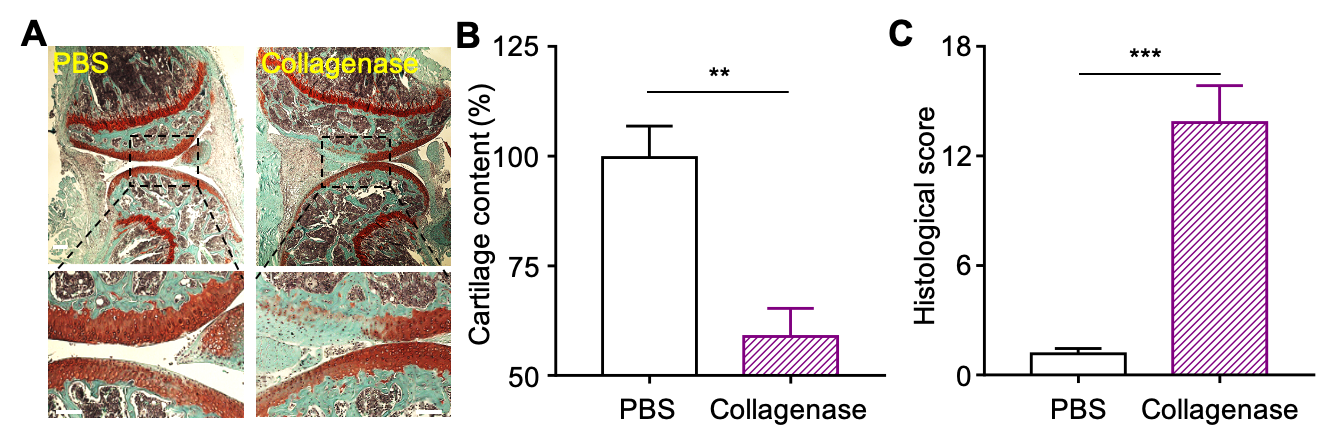


**Figure S3.** OA induction by collagenase in the knee joint of mice. (**A**) Representative images of safranin-O staining on cartilage sections upon PBS and collagenase treatment (intra-articular injection, 10 μL, 1 U) of healthy mice on day 0 and day 2. The mice were sacrificed on day 13 to collect knee joints for histopathological analysis. Scale bars, 100 μm. (**B**) Quantification of cartilage content from safranin-O-stained sections (red) in different groups. (**C**) Evaluation of cartilage damage through the histological scores based on typically OARSI scoring method. Data presented as mean + s.d. (*n* = 3); ***p* < 0.01, ****p* < 0.001; statistical analysis by one-way ANOVA.
